# Glutamyl Phosphate Is an Activated Intermediate in Actin Crosslinking by Actin Crosslinking Domain (ACD) Toxin

**DOI:** 10.1371/journal.pone.0045721

**Published:** 2012-09-21

**Authors:** Elena Kudryashova, Caitlin Kalda, Dmitri S. Kudryashov

**Affiliations:** Department of Chemistry and Biochemistry, The Ohio State University, Columbus, Ohio, United States of America; University of Heidelberg Medical School, Germany

## Abstract

Actin Crosslinking Domain (ACD) is produced by several life-threatening Gram-negative pathogenic bacteria as part of larger toxins and delivered into the cytoplasm of eukaryotic host cells via Type I or Type VI secretion systems. Upon delivery, ACD disrupts the actin cytoskeleton by catalyzing intermolecular amide bond formation between E270 and K50 residues of actin, leading to the formation of polymerization-deficient actin oligomers. Ultimately, accumulation of the crosslinked oligomers results in structural and functional failure of the actin cytoskeleton in affected cells. In the present work, we advanced in our understanding of the ACD catalytic mechanism by discovering that the enzyme transfers the gamma-phosphoryl group of ATP to the E270 actin residue, resulting in the formation of an activated acyl phosphate intermediate. This intermediate is further hydrolyzed and the energy of hydrolysis is utilized for the formation of the amide bond between actin subunits. We also determined the pH optimum for the reaction and the kinetic parameters of ACD catalysis for its substrates, ATP and actin. ACD showed sigmoidal, non-Michaelis-Menten kinetics for actin (K_0.5_ = 30 µM) reflecting involvement of two actin molecules in a single crosslinking event. We established that ACD can also utilize Mg^2+^-GTP to support crosslinking, but the kinetic parameters (K_M_ = 8 µM and 50 µM for ATP and GTP, respectively) suggest that ATP is the primary substrate of ACD *in vivo*. The optimal pH for ACD activity was in the range of 7.0–9.0. The elucidated kinetic mechanism of ACD toxicity adds to understanding of complex network of host-pathogen interactions.

## Introduction

Actin is a ubiquitous and highly conserved eukaryotic protein intrinsically involved in numerous vital processes in living cells. Many pathogenic bacteria, viruses, and parasites evolved to produce highly specific toxins that selectively target host’s actin cytoskeleton to disrupt its cellular functions and/or usurp actin to pathogens’ benefit. Among those toxins is the Actin Crosslinking Domain (ACD). ACD was first discovered as a part of Multifunctional Autoprocessing Repeats-in-Toxin **(**MARTX) [1] and VgrG1 [2] toxins of *Vibrio cholera* and later found in MARTX toxins of Gram-negative pathogenic bacteria *Vibrio vulnificus* [3] and *Aeromonas hydrophila* [4]. Depending on the carrier toxin, ACD can be delivered to the cytoplasm of the host cell via either Type I (MARTX) [5], [6] or Type VI (VgrG1) [2] secretion systems. Both ACD containing toxins of *V. cholerae* contribute to inflammatory diarrhea, prolonged colonization of the small intestine and lethality in experimental mouse models [7]–[10], suggesting that ACD has generally evolved as a factor compromising the host’s immune system.

The only known target of ACD in the host cell is actin. ACD catalyzes a covalent crosslinking of monomeric actin to yield oligomers of various sizes [11]. We have shown that the energy of Mg^2+^-ATP is required for this reaction [12] and that the resulting oligomers are crosslinked via an amide bond between the K50 and E270 residues on adjacent actin subunits [13]. Because the K50 and E270 residues are separated by ∼ 20 Å in actin filament [14]–[17], zero-length crosslinking by ACD sterically interferes with actin polymerization, ultimately resulting in actin cytoskeleton disassembly and cell rounding [11].

ACD of MARTX and VgrG1 toxins have no significant sequence homology to other proteins and the structure of the enzyme is not known. However, a mutagenesis-based analysis of residues substantial for catalysis revealed distant homology of the ACD active site to that of the glutamine synthetase (GS) family of enzymes [18]. GS enzymes are ligases that utilize ATP to activate the γ-carboxyl group of glutamate via formation of a high-energy intermediate glutamyl phosphate [19]. Upon a nucleophilic attack by ammonia, γ-glutamyl phosphate forms a transition-state complex that, after a subsequent release of the phosphate group, results in the formation of glutamine [20]. Because the outcome of the reaction catalyzed by GS enzymes resembles the ACD-catalyzed actin crosslinking (except in that the substrates of ACD are large protein molecules), it was speculated that these groups of enzymes may utilize a similar enzymatic mechanism (18).

Mutation analysis of ACD revealed that only a small subset of residues critical for the GS catalyzed reactions are conserved in ACD (18). These conserved residues, that are indispensable for actin crosslinking by ACD, correspond to those that are involved in the coordination of both Mg^2+^ and ATP in the GS catalytic site [18]. Neither the analogous residues implicated in binding to the nucleotide alone, nor those interacting with the substrates glutamine and ammonia in GS, are essential for ACD catalysis, suggesting that the mechanisms of the two reactions may differ substantially and, therefore, call for in depth investigation.

In the present study, we have employed mutagenesis on actin and radioactive [γ-^32^P]ATP tracing to decipher the mechanism by which energy of ATP is utilized for actin crosslinking by ACD. In addition, we analyzed kinetic parameters of the actin crosslinking reaction catalyzed by *V. cholerae* ACD. The determined kinetic characteristics imply that ACD should be well saturated by the cellular concentrations of Mg^2+^-ATP; and physiological fluctuations in the cellular concentrations of monomeric actin may have only a minor influence on the crosslinking rate. Our results demonstrate that GTP, when present at physiologically relevant concentrations, can also be utilized by ACD to fuel the crosslinking reaction. Importantly, we discovered that the thermodynamically unfavorable formation of an amide bond between actin subunits is coupled to the transfer of the γ-phosphoryl group from Mg^2+^-ATP/GTP to E270 residue. We found that the hydrolysis of this acyl phosphate derivative is linked to the covalent crosslinking of the activated E270 to the K50 residue of another actin molecule.

## Materials and Methods

### Actin Purification

Skeletal muscle actin (Ca^2+^-ATP G-actin) was prepared from acetone powder from frozen rabbit skeletal muscles (obtained from Pel-Freez Biologicals) as described [21] and stored in G-buffer (5.0 mM TRIS, pH 8.0, 0.2 mM Ca^2+^-ATP, 5.0 mM β-mercaptoethanol) on ice for 2 weeks or flash frozen in liquid nitrogen for prolonged storage.

Construction of E270Q, E270D and K50C yeast actin mutants was described previously [13]. Mutant actins were purified using DNase I affinity chromatrography as described [22], [23].

### Recombinant Protein Expression and Purification

ACD plasmid encoding residues 1965–2409 of *V. cholera* MARTX toxin was a gift from Dr. Satchell (Northwestern University). Recombinant ACD and gelsolin segment 1 were expressed in the BL21(DE3) strain of *E.coli* (Novagen, EMD Millipore) and purified as described [11], [24].

### Preparation of Nucleotide-free Actin and AMP-PNP-actin

Nucleotide-free actin was prepared as described previously [25]. To prepare AMP-PNP-actin, Ca^2+^-ATP G-actin was treated twice with Dowex 1×2, 50–100 mesh ion exchange resin (Acros Organics) (25% bed-volume to sample volume) to remove free nucleotides [25]. The remaining ATP bound to actin was hydrolyzed to ADP by adding hexokinase (20 units/ml) and D-glucose (2.0 mM) and incubating for 30 minutes on ice. ADP in the actin nucleotide cleft was then substituted with a non-hydrolysable ATP analog, adenosine 5′-(β,γ-imido)triphosphate (AMP-PNP), by adding a 20 fold molar excess (0.5 mM) of the latter and incubating for additional 30 minutes on ice. Actin with AMP-PNP in the nucleotide cleft was then passed through a gel filtration spin column (Zeba; Invitrogen) equilibrated in nucleotide-free buffer to remove free nucleotides and dextrose. Immediately after, Latrunculin B was added and actin was supplemented with 2.0 mM MgCl_2_.

### ACD-catalyzed Crosslinking

Unless specified otherwise, ACD-catalyzed crosslinking was performed in a buffer containing 2.0 mM MgCl_2_, 0.2 mM EGTA, 0.5 mM ATP and either 10 mM HEPES, pH 7.5 or 10 mM TRIS, pH 8.0. Actin was pre-incubated with 1.5 molar excess of either Latrunculin B or gelsolin segment 1 for at least 15 minutes prior to initiation of crosslinking to prevent polymerization and inhibit nucleotide exchange. ACD is inactive in the absence of Mg^2+^ [11], therefore, crosslinking was initiated by either adding ACD (0.025–0.05 µM) to actin containing MgCl_2_, or by addition of MgCl_2_ (2.0 mM) to a mixture of actin and ACD. All reactions were performed at 24°C and the crosslinking was stopped within 1–3 minutes of initiation by adding SDS-PAGE sample buffer. Crosslinked actin species were resolved on 7.5% SDS-gels and stained with Coomassie Brilliant Blue R-250. Densitometry was performed using Perfection V600 EPSON scanner and ImageJ image-processing software (http://rsb.info.nih.gov/ij/). The rates of ACD activity in a single experiment were expressed in µmoles of bonds formed per minute per µmole of the enzyme.

### pH Dependence of ACD Activity

The pH dependence of ACD activity was assessed using 50 mM solutions of the following buffers: citrate pH 5.0; MES pH 5.0–6.0; MOPS pH 7.0–8.0; HEPES pH 7.0–8.0; TRIS pH 7.0–9.0; TRICINE pH 8.0; TAPS pH 8.0–9.0; glycine pH 9.0–11.0; and universal Teorell-Stenhagen buffer over a broad range of pH [26].

### Kinetic Parameters of ACD Crosslinking

All kinetic analyses were performed with purified recombinant ACD. Purity of the isolated ACD was higher than 95% as determined by densitometry of Coomassie-stained SDS-gels. Kinetic parameters of ACD for actin were measured in the presence of increasing concentrations of actin (0–100 µM) complexed with Latrunculin B at constant concentration of ATP (1.0 mM). To determine kinetic parameters of ACD for ATP and GTP, the reactions were carried out with saturating concentrations of actin (100 µM) in the presence of gelsolin segment 1 (120 µM) to prevent ATP release from the nucleotide-binding cleft of actin. The actin/gelsolin segment 1 mixture was passed through two cycles of 15-minute incubation with Dowex resin to remove free ATP from the solution. Increasing concentrations of the nucleotides (GTP or ATP) were added and the crosslinking reaction was initiated by the addition of 2.0 mM MgCl_2_. The initial rates of ACD activity were plotted *versus* actin or nucleotide concentrations; and the K_M_/K_0.5_ and K_cat_ values were determined by fitting the experimental curves to a classical or modified (Equation 2) Michaelis-Menten equation.

### Growth Curves of Yeast Actin Mutants

*S. cerevisiae* strains expressing wild type (WT) or E270Q, E270D and K50C actin mutants were grown from fresh colonies in YPD medium overnight. The cell suspensions were diluted to 0.1 A_600_ and grown in a 96-well plate (Greiner) covered with Thermal Seal RT2 film (Bioexpress) punched with a needle for aeration. Cell densities (A_600_) were monitored by Infinite M1000Pro (Tecan Group Ltd.) plate reader using the following parameters: temperature –30°C; kinetic cycle (duration) –12 h; kinetic intervals –30 min; absorbance – wavelength 600 nm, 25 flashes, 100 ms settle time; orbital shaking – twice for 890 s, with amplitude 6 mm, 120 rpm. Growth curves were plotted as A_600_ *versus* time.

### Autoradiographic Analysis

5 µM K50C and 10 µM E270Q and E270D yeast actin mutants were incubated with 0.1 µM ACD in the presence of 2.0 mM MgCl_2_ and 50 µM (40 pCi/µl) of [γ-^32^P]ATP. Reactions were stopped by the addition of SDS-PAGE sample buffer and the proteins were loaded promptly and resolved by SDS-gel electrophoresis. Gels were rinsed, dried between layers of cellophane film in a BioRad gel-drying apparatus, and exposed to phosphor screens (Molecular Dynamics). After 4–16 h exposure, the screens were scanned and analyzed using a variable scanner Typhoon Trio (GE Healthcare). When desired, dry gels were soaked in water, separated from cellophane and stained with Coomassie Brilliant Blue R-250.

**Figure 1:**
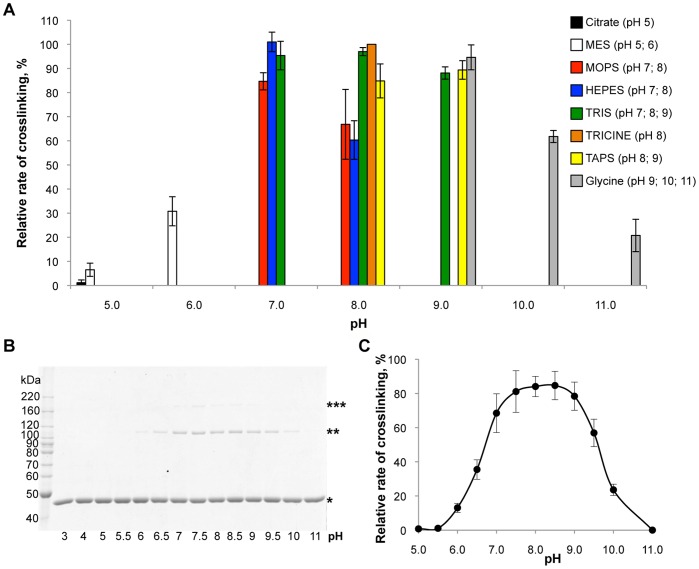
pH-dependence of the ACD activity. (A) Crosslinking of rabbit skeletal actin (5.0 µM) by ACD (0.025 µM; 1∶200 molar ratio to actin) was assessed in 50 mM buffers of various pH and plotted against the corresponding pH values. The initial rates of ACD activity were normalized and expressed as percent of the reaction rate in TRICINE buffer at pH 8.0. (B) Representative SDS-gel of actin crosslinking in the Teorell-Stenhagen buffer system at various pH values. Single, double, and triple asterisks denote actin monomer, dimer, and trimer, respectively. (C) Quantitation of ACD activity in universal Teorell-Stenhagen buffer system reveals typical bell-shaped pH dependence. Error bars in A and C represent standard errors of means; n = 3.

## Results

### pH-dependence of the ACD Activity

The initial rate of actin crosslinking by ACD was analyzed using a variety of buffers in the pH range of 5.0–11.0. ACD activity exhibited a typical bell-shaped pH dependence profile with broad optimum at pH 7.0–9.0, decreasing to 35% of the peak activity at pH 6.5 and to 55% at pH 9.5 (Figure 1). Therefore, the pH optimum for ACD is well within the physiological range of cytosolic pH.

**Figure 2:**
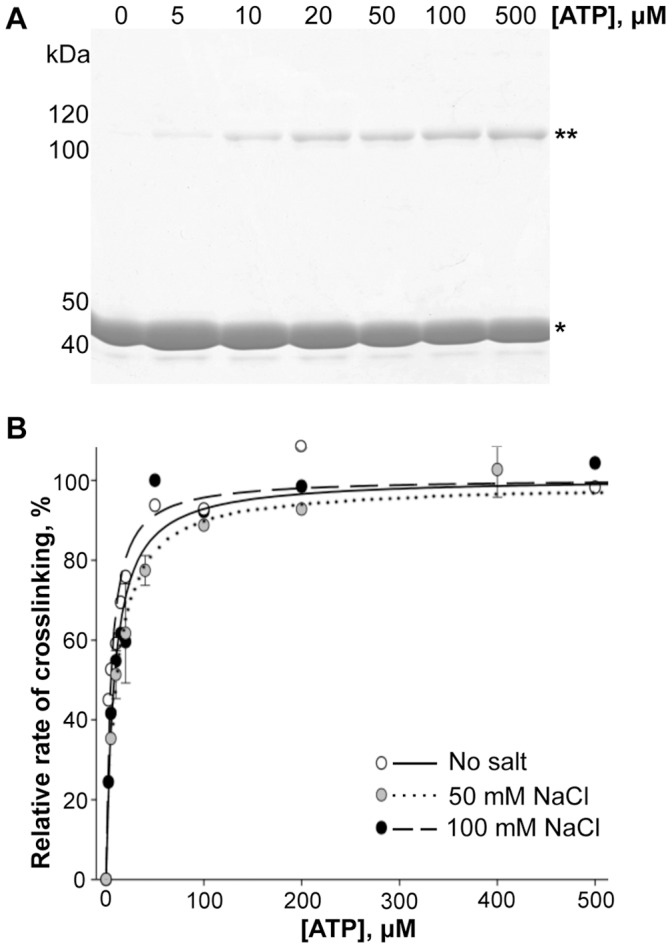
Kinetic parameters of actin crosslinking by ACD for ATP. (A) SDS-gel analysis of ACD-catalyzed crosslinking of rabbit skeletal actin (100 µM) in the presence of increasing concentrations of ATP. Single and double asterisks denote actin monomer and dimer, respectively. (B) The initial rates of actin crosslinking were plotted *versus* increasing concentrations of ATP (0–1000 µM). ACD activity was normalized in all experiments and expressed in percent of K_cat_ for each reaction. Lines represent fits to classical Michaelis-Menten equation. Error bars represent standard errors of means.

### Kinetic Parameters of Actin Crosslinking with ACD for ATP

Michaelis-Menten constants of enzyme/substrate pairs determined under carefully controlled *in vitro* conditions provide valuable information for understanding the efficiency of catalysis under *in vivo* conditions. The catalysis by ACD is a three-substrate enzymatic reaction that involves Mg^2+^-ATP and two actin molecules. The determination of the K_M_ of ACD for ATP requires the nucleotide to be present in a wide range of concentrations while the concentration of another substrate, actin, must saturate the enzyme. This is a technically challenging task because ATP is an integral part of actin structure that defines its conformational stability [25], [27], and therefore must be supplied together with actin in the bound state. We have shown previously that in the absence of free ATP in solution, ACD can efficiently utilize ATP leaking from the complex [12]. To circumvent this obstacle, we employed the ability of gelsolin segment 1 to block the nucleotide exchange from the actin cleft upon interaction with actin [12], [28]. Free ATP was then completely removed from the solution via two cycles of incubation with Dowex ion exchange resin [25].

Under near-saturating concentrations of actin, the initial rates of crosslinking plotted *versus* concentrations of ATP had typical hyperbolic Michaelis-Menten dependence (Figure 2) with a K_M_ of 7.8±1.4 µM in the absence of salt (Table 1). Changing salt conditions did not significantly affect the K_M_ (7.2±1.0 µM in the presence of 100 mM NaCl; Table 1), suggesting that the binding of the nucleotide to ACD is not substantially altered by the ionic strength. Due to the intrinsic instability of ACD, the K_cat_ of the reaction varied significantly between different experiments and enzyme preparations and therefore measurements had to be conducted simultaneously to gain quantitatively comparable data. In a representative experiment, the K_cat_ dropped from 449.2±17.7 down to 223.2±5.2 µmole bonds×min^−1^×µmole^−1^ of ACD in the absence and in the presence of 100 mM NaCl, respectively (Table 1).

**Table 1:** Kinetic parameters of ACD for ATP.

Salt concentration	K_cat_ *	K_M_ (µM)
No salt	449.2±17.7	7.8±1.4
100 mM NaCl	223.2±5.2	7.2±1.0

* K_cat_ is expressed in µmole bonds×min^−1^×µmole^−1^ of enzyme.

K_M_ and K_cat_ values were determined by fitting the initial rates of accumulation of actin oligomers (dimers and trimers) to classical Michaelis-Menten equation; errors were obtained from least squares analyses of the fits.

### GTP as a Substrate for ACD

**Figure 3:**
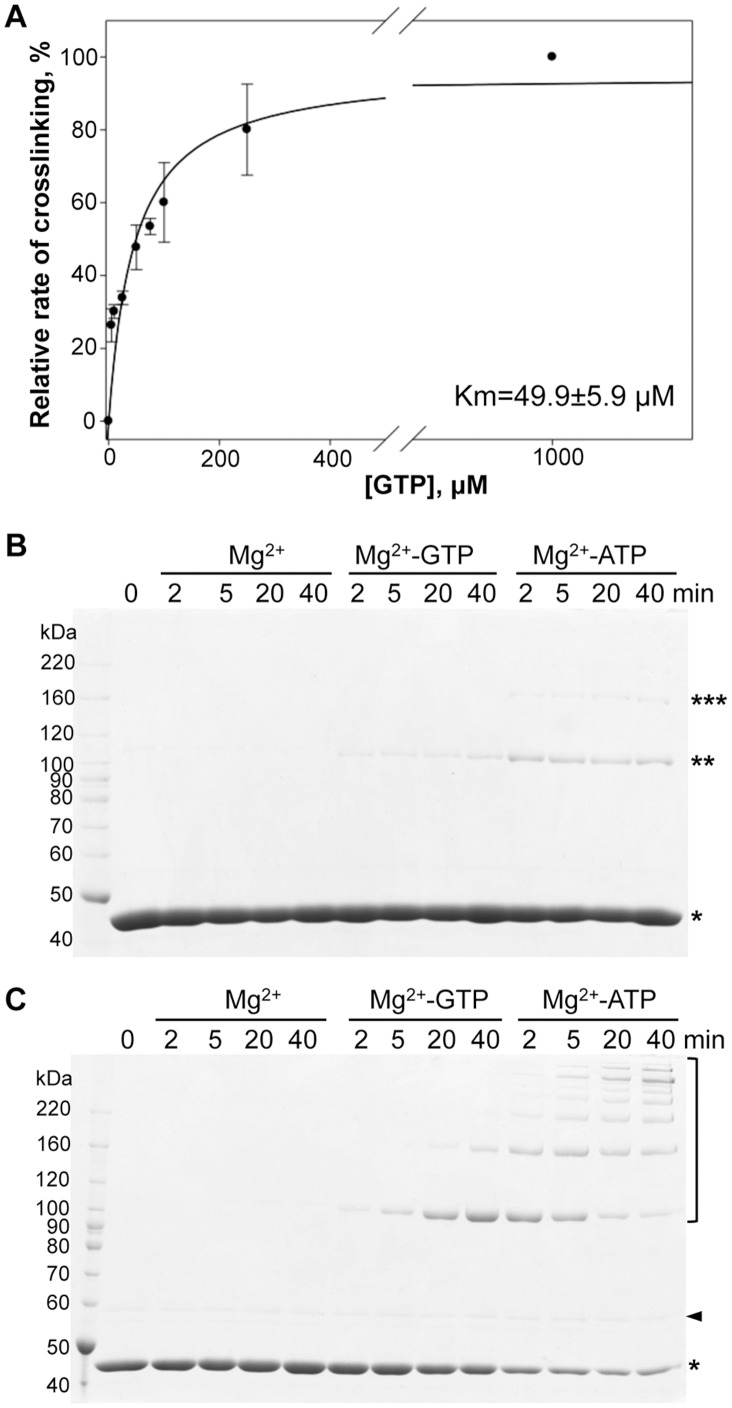
GTP is a substrate for ACD. (A) ACD activity rates were determined and plotted *versus* increasing GTP concentrations (0–1000 µM). Lines represent fits to classical Michaelis-Menten equation. Error bars represent standard errors of means; n = 2. (B) SDS-gel analysis of ACD-catalyzed crosslinking of nucleotide-free actin (30 µM) in the absence of free nucleotides or in the presence of 1.0 mM of either GTP or ATP. Single, double, and triple asterisks denote actin monomer, dimer, and trimer, respectively. (C) SDS-gel analysis of ACD-catalyzed crosslinking of AMP-PNP-actin (20 µM) in the absence of free nucleotides or in the presence of 1.0 mM of either GTP or ATP. Asterisk denotes actin monomer, arrowhead – hexokinase, bracket – crosslinked actin species. Note that no crosslinking of actin was observed in the absence of ATP or GTP added both in (B) and (C). Limited extent of crosslinking in the presence of GTP reflects a lower K_cat_ and higher K_M_ of ACD for this substrate. Rabbit skeletal actin was used in (A)–(C).

With cellular concentrations in the range of 0.1–0.5 mM [29], GTP is the second most abundant energy molecule in the cell after ATP and the most structurally similar to ATP nucleoside triphosphate. Therefore, we tested the ability of ACD to utilize GTP as a source of energy for actin crosslinking. In the experimental setup similar to one described above for ATP, we found that GTP can fuel the crosslinking reaction catalyzed by ACD with a mean K_M_ of 49.9±5.9 µM (Figure 3A). K_cat_ was determined to be 26.6±2.7 µmole bonds×min^−1^×µmole^−1^ of ACD.

In this experiment the nucleotide release from the actin cleft was blocked with gelsolin segment 1, and free ATP was removed from the solution before the addition of GTP. If GTP from solution could replace ATP in the nucleotide cleft of actin, this would cause an influx of ATP into the solution making it available for ACD. This would generate ambiguity in the interpretation of our results. However, the above scenario is unlikely given that the affinity of actin to GTP is ∼500 to 12,000 times lower than to ATP [30], [31]. Nonetheless, we eliminated any potential uncertainty by completely removing ATP from actin using two separate approaches. In the first set of experiments we used an established method to prepare nucleotide-free actin by hydrolyzing all ATP to AMP with apyrase in the presence of 50% sucrose to protect actin from denaturation [25]. Using nucleotide-free actin as a substrate for ACD, we observed the accumulation of crosslinked actin species upon addition of either ATP or GTP, but not in the absence of the nucleotides (Figure 3B). The activity of ACD in this experiment was low and the accumulation of oligomers ceased within two minutes, likely due to the rapid decomposition of extraneously added nucleotides by apyrase. Therefore, in the second set of experiments ATP was hydrolyzed to ADP with hexokinase, the activity of which depends on the presence of D-glucose and can therefore be easily controlled. To further eliminate possible traces of ATP, we replaced ADP in the nucleotide binding cleft of actin with AMP-PNP and removed the excess of the latter from the solution. Addition of Mg^2+^-GTP to AMP-PNP-actin supported the crosslinking of actin, albeit less efficiently than the addition of Mg^2+^-ATP (Figure 3C). In both experiments (Figures 3B and 3C) the lack of actin crosslinking by ACD in the absence of the extraneous nucleotides confirms the complete removal of ATP from actin. Altogether, these experiments explicitly demonstrate that GTP is an alternative substrate for ACD.

### Kinetic Parameters of Actin Crosslinking with ACD for Actin

**Figure 4:**
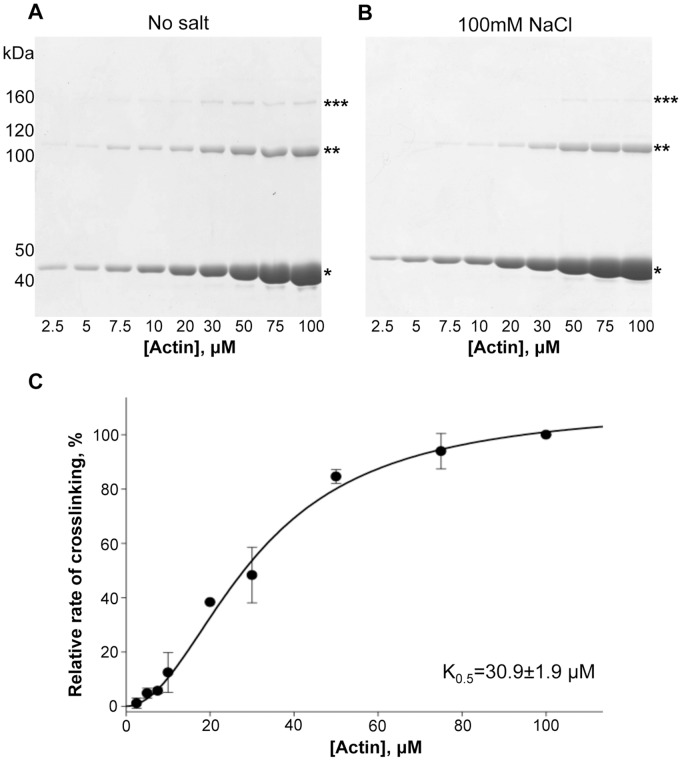
Kinetic parameters of rabbit skeletal actin crosslinking with ACD for actin. (A) and (B) Representative SDS-gels of ACD crosslinking reactions with increasing concentrations of actin (2.5–100 µM) in the absence (A) or presence of 100 mM NaCl (B). (C) The initial rates of actin crosslinking by ACD were plotted *versus* increasing concentrations of actin. Plot shows composite normalized rate of ACD activity *versus* actin concentrations attained at different salt conditions (0 and 100 mM NaCl). Lines represent fits to modified Michaelis-Menten equation (Equation 2). Error bars represent standard errors of means; n = 4.

In contrast to a hyperbolic dependence of the initial crosslinking rates on the concentrations of ATP and GTP, the rate of crosslinking has a sigmoidal non-Michaelis dependence on the concentration of actin (Figure 4). This result is in agreement with our previous finding that actin participates in the crosslinking reaction as two different substrates: one actin molecule is the donor of the side chain carboxyl group of E270 and another molecule is the donor of the primary amine group of K50 [13]. These residues are located respectively in the hydrophobic and the DNase I loops, which are separated on the actin molecule by ∼46 Å. Therefore, by varying actin concentration we simultaneously change the concentration of both substrates. In a simplified form, the crosslinking of two actin molecules by ACD can be described as follows:
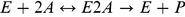
(1)where *E* is ACD, *A* is actin, and *P* is a crosslinked actin dimer. A re-derivation of the Michaelis-Menten equation under these conditions yields a modified expression, identical in appearance to a Hill equation:
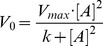
(2)It is easy to recognize that 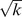 has the physical meaning of the actin concentration at half-maximal rate (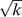 = K0.5). The experimental curves fit well to this equation, resulting in a mean K0.5 of 30.9±1.9 µM (Figure 4). Typical Kcat and K0.5 data obtained in a representative experiment under different salt conditions are shown in Table 2. The K0.5 of ACD for actin is at or below the concentration of G-actin in most eukaryotic cells [32], suggesting that ACD activity is close to optimal under wide range of cellular conditions.

**Table 2:** Kinetic parameters of ACD for actin under various salt conditions.

Salt concentration	K_cat_ *	K_0.5_ (µM)
No salt	531.2±62.0	26.0±5.6
100 mM NaCl	382.3±78.8	40.2±3.4
100 mM KCl	162.6±28.6	35.6±3.7

* K_cat_ is expressed in µmole bonds×min^−1^×µmole^−1^ of enzyme.

K_0.5_ and K_cat_ values were determined by fitting the initial rates of accumulation of actin oligomers (dimers and trimers) to modified Michaelis-Menten equation (Equation 2); errors were obtained from least squares analyses of the fits.

### Formation of Glutamyl Phosphate is an Intermediate Step of the Catalysis

Energetically unfavorable formation of an amide bond between carboxyl and amine groups is typically coupled to the splitting of an ATP phosphoanhydride bond resulting in the activation of a carboxyl group via attachment of inorganic phosphate, pyrophosphate, or AMP [33]. We have shown previously that hydrolysis of Mg^2+^-ATP is required for actin crosslinking by ACD and that inorganic phosphate (P_i_) is released in the course of amide bond formation between inter-strand E270 and K50 actin residues [12], [13]. Therefore, mutagenesis on E270 and K50 residues of actin allows the separation of the donors of the carboxyl and amine groups into two discrete protein entities.

Since actin is an essential protein for yeast, functional performance of yeast actin mutants *in vivo* can be evaluated by analyzing the growth rate of yeast strain that expresses a mutant as its only actin. We analyzed the growth of yeast strains expressing K50C, E270Q and E270D actin mutants to test their ability to substitute WT actin in yeast cell growth. *S. cerevisiae* strains expressing K50C and E270Q showed growth rates comparable to that of the WT strain, whereas the E270D expressing cells plateaued at a lower density (Figure S1). Therefore, the K50C and E270Q actin mutants effectively replace WT actin in yeast cells suggesting that these mutations do not substantially affect the structure and functions of actin.

**Figure 5:**
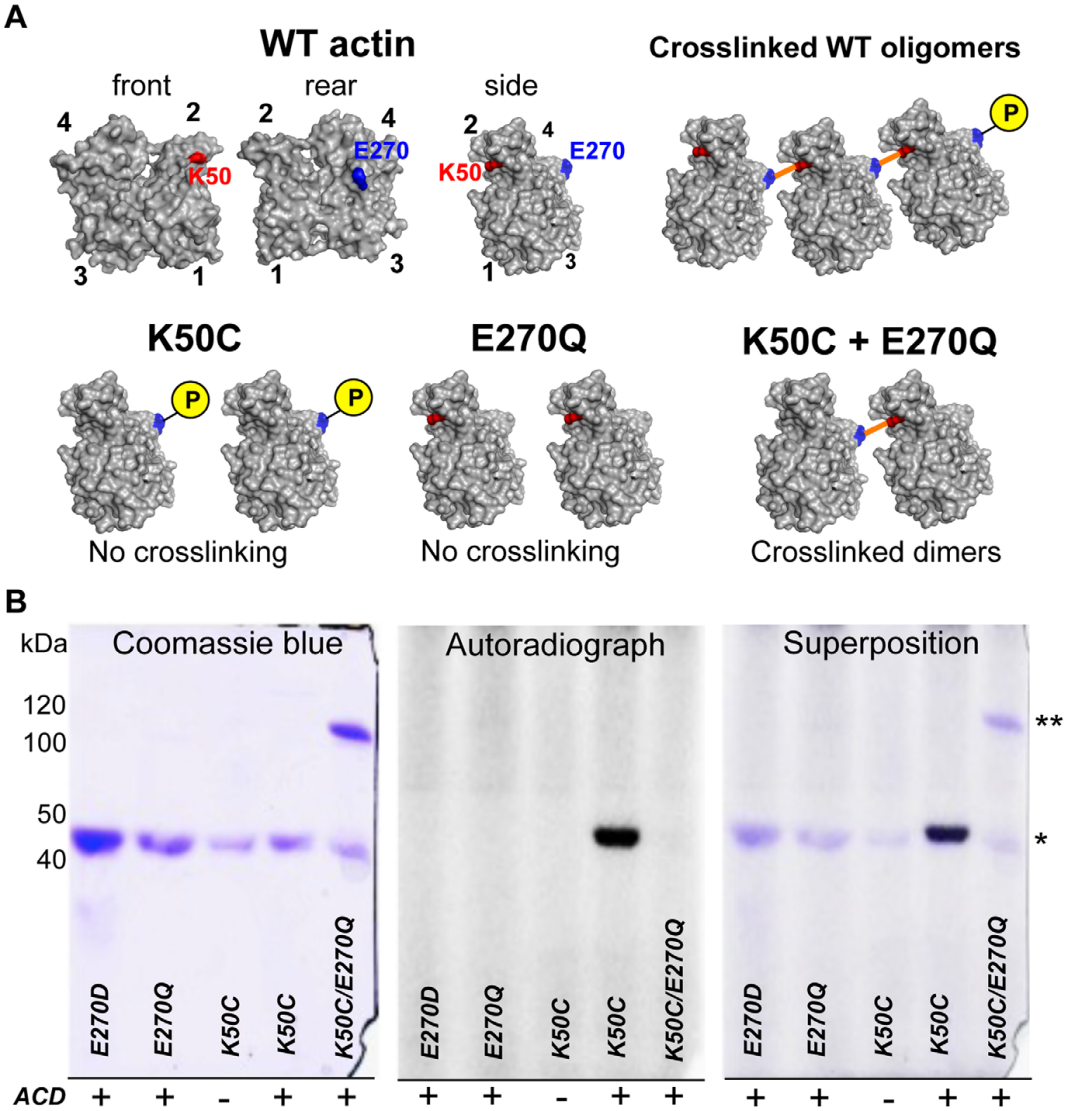
Formation of glutamyl phosphate is intermediate, activation step of the ACD catalysis. (A) Schematic representation of the ACD-catalyzed crosslinking of actin mutants. WT actin, as well as K50C, and E270K actin mutants are represented as surface contour images with intact K50 and E270 residues colored in red and blue, respectively; actin subdomains are numbered 1–4. Crosslinking of WT actin produces chains of oligomers, individual K50C and E270Q mutants produce no crosslinking, whereas their mixture produces dimers only. (B) Crosslinking of K50C and E270Q or E270D yeast actin mutants in the presence of γ-[^32^P]ATP was assessed by SDS-PAGE (left panel) and autoradiography (middle panel); right panel represents the merged image of the Coomassie-stained gel and the autoradiograph. To ensure the complete crosslinking of K50C mutant (5 µM), double amount of E270Q mutant (10 µM) was added; therefore, the remaining monomer in lane 5 of the Coomassie-stained gel is E270Q actin. Notably, γ-[^32^P]P_i_ is incorporated only into K50C mutant (lane 4) and is released upon its crosslinking to dimer (lane 5).

To track the fate of inorganic phosphate that is released in the course of crosslinking, we conducted the ACD catalyzed reaction with purified K50C, E270Q, and E270D actin mutants in the presence of radioactive [γ-^32^P]ATP. In agreement with our previous report [13], neither of the individual mutants produced crosslinked species in the presence of ACD, whereas mixing of two individual mutants together in the same reaction resulted in the formation of a dimer, but not of higher crosslinked species (Figure 5). Most importantly, autoradiographic analysis of SDS-gels showed incorporation of radioactive [γ-^32^P]P_i_ into the K50C actin mutant, but not in E270Q or E270D mutants (Figure 5B). This data suggest that ACD specifically catalyzes the transfer of P_i_ from ATP to intact E270, whereas even conservative substitution of glutamate to aspartate completely abolishes the translocation of the phosphoryl group.

**Figure 6:**
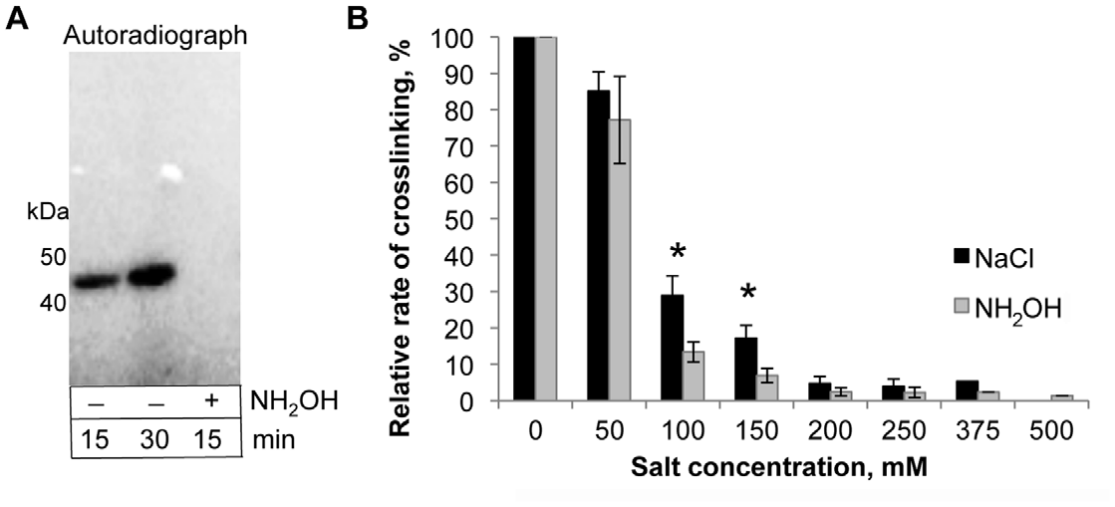
γ-[^32^P]-phosphate removal by hydroxylamine. (A) K50C yeast actin was phosphorylated at E270 by ACD, treated with 100 mM hydroxylamine, and analyzed by autoradiography. Note that γ-[^32^P]P_i_ was completely removed from K50C after incubation with hydroxylamine. (B) Inhibition of ACD crosslinking activity by hydroxylamine or NaCl. Error bars represent standard errors of means; n = 5. Statistical analysis was carried out by 2-tailed Student’s *t* test (*−*p*<0.05).

Hydroxylamine is not reactive towards phosphorylated Ser, Thr, or Tyr residues, but selectively reacts with acyl-phosphates with a formation of acyl hydroxamate [34]. To verify that the phosphorylation occurs at an acyl (glutamyl) residue, hydroxylamine was added to K50C actin that was pre-incubated with ACD for 15 minutes and then denatured with SDS sample buffer. [γ-^32^P]P_i_ was completely removed from actin after 15 min incubation in the presence of 100 mM hydroxylamine as confirmed by SDS-gel radiography analysis (Figure 6A). Furthermore, under native conditions hydroxylamine also inhibited crosslinking of actin by ACD (Figure 6B), albeit to a limited degree.

## Discussion

In the present work, we established the mechanism by which the energy of ATP is utilized for actin crosslinking by the *V. cholerae* ACD toxin and determined the kinetic parameters and pH optimum for this reaction.

Our findings provide evidence that ACD utilizes the energy of Mg^2+^-ATP/GTP for activation of the E270 residue of actin. This activation occurs via formation of a high-energy glutamyl phosphate intermediate, which is subsequently hydrolyzed upon formation of an amide bond with the K50 residue of another actin molecule. This was confirmed by the observed removal of the radioactive phosphate from E270 of one actin monomer (K50C mutant) upon its crosslinking to the K50 residue of another actin molecule (E270Q mutant) (Figure 5B). A similar mechanism is utilized by the GS family of enzymes, which activate glutamic acid via formation of a high-energy acyl phosphate intermediate [35], [36] and whose active sites share a distant sequence similarity with the putative ACD catalytic center [18]. Therefore, ACD is a ligase that first phosphorylates one actin subunit and then crosslinks it to another subunit.

Hydroxylamine-mediated removal of the incorporated [γ-^32^P]-phosphoryl group under denaturing conditions further confirmed that phosphorylation occurs at an acyl residue. However under native conditions, the hydroxylamine-induced inhibition of the ACD-catalyzed reaction was only slightly more efficient than that caused by NaCl (Figure 6). This observation suggests that most of the observed inhibition originated from the ionic strength of the hydroxylamine/HCl solution, which was neutralized by the addition of near-equal concentration of NaOH. We speculate that the poor inhibitory effect of hydroxylamine under native conditions reflects limited exposure of the phosphorylated E270 to solvent during catalysis. This likely implies that the crosslinking reaction proceeds via formation of a ternary complex between the enzyme and two actin molecules.

The pH optimum for the ACD enzymatic activity (pH 7.0–9.0) covers the entire physiological range of the cytosolic pH; but lower activity of ACD can be expected under conditions of cellular acidosis. In general, a pH-dependence of enzymatic reactions reflects the ionization state of amino acid residues directly involved in catalysis and/or interaction with a substrate [26]. The sharp drop of the rate of catalysis at pH below 7, as observed in our experiments (Figure 1), may reflect changes in the ionization state of histidine residues at the catalytic site, which agrees with the finding that His2083 (numeration of the MARTX*_Vc_* sequence) plays important role in the ACD activity [18].

The enzymatic reaction catalyzed by ACD involves three substrates: one molecule of Mg^2+^-ATP and two molecules of actin. Given that the cellular ATP concentration (0.5–10 mM) [29] well exceeds the K_M_ of the enzyme for this substrate determined in the present study (8 µM), we predict that ACD is generally well saturated by ATP under a wide range of cellular conditions. Our results indicate that ACD can also utilize GTP as a source of energy for the actin crosslinking reaction (Figure 3). However, considering substantially lower cellular concentration of GTP and given that the K_M_ of ACD for GTP (50 µM) is about 6 times higher and the K_cat_ is almost 17 times lower than those parameters observed for ATP, we can predict that GTP is only a secondary substrate for ACD under physiological conditions.

Interestingly, the K_M_ of ACD for ATP is at least several times lower than those determined for GSs of various origins (33 µM –1.85 mM; BRENDA - BRaunschweig ENzyme Database http://www.brenda-enzymes.info/) [37]. Therefore, it is reasonable to speculate that the high affinity of ACD for ATP is an essential element of the pathogenic efficiency of this toxin under a variety of cellular conditions.

The other substrate of ACD is actin. Notably, two actin monomers participate in the crosslinking reaction as two different substrates: i) the donor of the side chain carboxyl group of E270 and ii) the donor of the side chain primary amine group of K50 [13]. Although Equation 2 does not specify whether actin molecules bind to the enzyme simultaneously, independently, or sequentially, it provides a good fit for the experimental data (within the 95% confidence interval) and allows the calculation of the half-saturating concentration of actin for the crosslinking reaction (K_0.5_ = 30 µM). These *in vitro* data agree with the inhibition of actin crosslinking in cells treated with a filament-stabilizing drug Dolastatin 11 and the potentiation of crosslinking by a G-actin-sequestering drug Latrunculin B [11]. The concentration of G-actin in most eukaryotic cells is in the range of 100 to 300 µM with only a few documented exceptions such as *S. cerevisae* cells (0.01 µM) and *Xenopus* oocytes (12 µM) [32]. Two major G-actin binding proteins in the cell - thymosin β4 and profilin - do not interfere with actin crosslinking by ACD [12], suggesting that the enzyme is typically saturated or nearly saturated by cellular levels of G-actin. To exemplify, we can predict that actin crosslinking by ACD will be very efficient in the phagocytic immune cells, which appear to be the primary target for ACD-containing VgrG1 toxin [10], as the concentration of monomeric actin in these cells is in the 160–300 µM range [32], [38].

A putative catalytic site of ACD, predicted based on a distant sequence similarity with glutamine synthetases, contains a number of glutamate and arginine residues as well as a histidine residue [18]. In addition, both actin residues involved in crosslinking (K50 and E270), are charged under neutral pH suggesting that ionic strength might have a significant impact on the reaction. Indeed, increasing ionic strength with either NaCl or KCl, affects both K_cat_ and K_0.5_ for actin by decreasing the former and moderately increasing the latter (Table 2), suggesting that the reaction is inhibited by salts via a complex mechanism that involves elements of competitive and non-competitive inhibition.

In summary, the results of this work revealed hitherto unknown details of the mechanisms of toxicity employed by life-threatening ACD-producing pathogenic bacteria: *Vibrio cholerae*, *Vibrio vulnificus,* and *Aeromonas hydrophila*. We found that the ACD-catalyzed actin crosslinking proceeds via phosphorylation-mediated activation of the E270 residue on one actin subunit coupled with its subsequent linkage to the K50 residue on another actin molecule. Furthermore, the kinetic parameters of ACD catalysis determined *in vitro* predict that ACD toxins exhibit optimal activity under the physiological pH, nucleotide, and G-actin concentrations. Therefore, the present study elucidates a highly unusual mechanism of toxicity employed by the ACD toxins and thereby sets the conditions for future interventions against this family of enzymes, and for the development of ACD-based crosslinking tools for cytoskeleton studies.

## Supporting Information

Figure S1 **Growth curves of WT and K50C, E270Q, and E270D yeast actin strains.** Growth of yeast strains expressing WT or mutated actin was monitored by A_600_ and plotted *versus* time. Error bars represent standard deviations of means of twelve replicates.
(TIFF)
