# Supplementary figures and images for: Glutamyl Phosphate Is an Activated Intermediate in Actin Crosslinking by Actin Crosslinking Domain (ACD) Toxin
Source: PLoS One. 2012 Sep 21;7(9):e45721. doi: 10.1371/journal.pone.0045721 (PMC3448709; doi:10.1371/journal.pone.0045721)

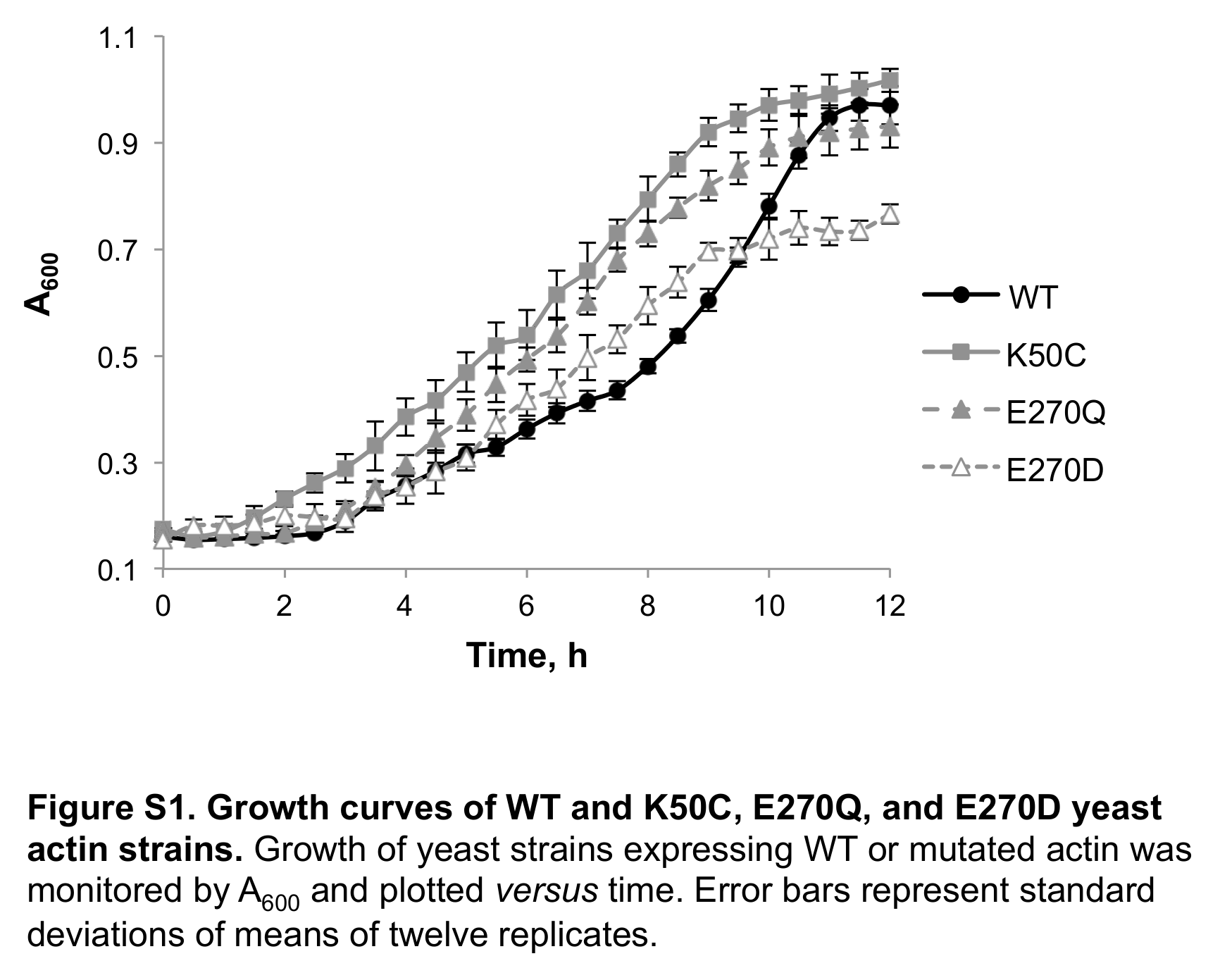

Supplement: Figure S1 — Growth curves of WT and K50C, E270Q, and E270D yeast actin strains. Growth of yeast strains expressing WT or mutated actin was monitored by A600 and plotted versus time. Error bars represent standard deviations of means of twelve replicates. (TIFF) [file pone.0045721.s001.tif]
